# A Soft Label Deep Learning to Assist Breast Cancer Target Therapy and Thyroid Cancer Diagnosis

**DOI:** 10.3390/cancers14215312

**Published:** 2022-10-28

**Authors:** Ching-Wei Wang, Kuan-Yu Lin, Yi-Jia Lin, Muhammad-Adil Khalil, Kai-Lin Chu, Tai-Kuang Chao

**Affiliations:** 1 Graduate Institute of Biomedical Engineering, National Taiwan University of Science and Technology, Taipei 106335, Taiwan; 2 Graduate Institute of Applied Science and Technology, National Taiwan University of Science and Technology, Taipei 106335, Taiwan; 3 Department of Pathology, Tri-Service General Hospital, Taipei 11490, Taiwan; 4 Institute of Pathology and Parasitology, National Defense Medical Center, Taipei 11490, Taiwan

**Keywords:** HER2 overexpression, fluorescence in situ hybridization, brightfield dual in situ hybridization, metastatic breast cancer, thyroid cancer, fine needle aspiration, thin prep, soft label deep learning

## Abstract

**Simple Summary:**

Early diagnosis and treatment of cancer is crucial for the survival of cancer patients. Pathologists can use computational pathology techniques to make the diagnosis process more efficient and accurate. With the emergence of deep learning, there is considerable hope that this technology will be able to address issues that were previously impossible to tackle. In this study, we present an automatic soft label deep learning framework to select patients for human epidermal factor receptor 2 target therapy and papillary thyroid carcinoma diagnosis. This approach will assist in breast cancer target therapy and thyroid cancer diagnosis with rapid examination and decrease human judgment mistakes.

**Abstract:**

According to the World Health Organization Report 2022, cancer is the most common cause of death contributing to nearly one out of six deaths worldwide. Early cancer diagnosis and prognosis have become essential in reducing the mortality rate. On the other hand, cancer detection is a challenging task in cancer pathology. Trained pathologists can detect cancer, but their decisions are subjective to high intra- and inter-observer variability, which can lead to poor patient care owing to false-positive and false-negative results. In this study, we present a soft label fully convolutional network (SL-FCN) to assist in breast cancer target therapy and thyroid cancer diagnosis, using four datasets. To aid in breast cancer target therapy, the proposed method automatically segments human epidermal growth factor receptor 2 (HER2) amplification in fluorescence in situ hybridization (FISH) and dual in situ hybridization (DISH) images. To help in thyroid cancer diagnosis, the proposed method automatically segments papillary thyroid carcinoma (PTC) on Papanicolaou-stained fine needle aspiration and thin prep whole slide images (WSIs). In the evaluation of segmentation of HER2 amplification in FISH and DISH images, we compare the proposed method with thirteen deep learning approaches, including U-Net, U-Net with InceptionV5, Ensemble of U-Net with Inception-v4, Inception-Resnet-v2 encoder, and ResNet-34 encoder, SegNet, FCN, modified FCN, YOLOv5, CPN, SOLOv2, BCNet, and DeepLabv3+ with three different backbones, including MobileNet, ResNet, and Xception, on three clinical datasets, including two DISH datasets on two different magnification levels and a FISH dataset. The result on DISH breast dataset 1 shows that the proposed method achieves high accuracy of 87.77 ± 14.97%, recall of 91.20 ± 7.72%, and F1-score of 81.67 ± 17.76%, while, on DISH breast dataset 2, the proposed method achieves high accuracy of 94.64 ± 2.23%, recall of 83.78 ± 6.42%, and F1-score of 85.14 ± 6.61% and, on the FISH breast dataset, the proposed method achieves high accuracy of 93.54 ± 5.24%, recall of 83.52 ± 13.15%, and F1-score of 86.98 ± 9.85%, respectively. Furthermore, the proposed method outperforms most of the benchmark approaches by a significant margin (*p* <0.001). In evaluation of segmentation of PTC on Papanicolaou-stained WSIs, the proposed method is compared with three deep learning methods, including Modified FCN, U-Net, and SegNet. The experimental result demonstrates that the proposed method achieves high accuracy of 99.99 ± 0.01%, precision of 92.02 ± 16.6%, recall of 90.90 ± 14.25%, and F1-score of 89.82 ± 14.92% and significantly outperforms the baseline methods, including U-Net and FCN (*p* <0.001). With the high degree of accuracy, precision, and recall, the results show that the proposed method could be used in assisting breast cancer target therapy and thyroid cancer diagnosis with faster evaluation and minimizing human judgment errors.

## 1. Introduction

Cancer is the largest cause of mortality in the world, accounting for over 10 million deaths in 2020. Early detection and treatment of cancer reduce deaths. However, the detection of cancer is one of the most difficult tasks in cancer pathology. Trained pathologists can analyze complicated tissue structures and detect tumors, but the judgements are subjective, qualitative, and time-consuming, resulting in significant intra- and inter-observer variability. Pathologists’ exhaustion and fatigue may contribute to diagnostic mistakes as workload increases, lowering the overall quality of pathology service. To deal with this problem, modern processing techniques such as artificial intelligence (AI) techniques have been developed. Deep learning (DL), a subset of AI capable of autonomously extracting valuable properties from images to achieve specified tasks, has been repeatedly shown to outperform standard image-processing algorithms, as demonstrated for image classification [1] or segmentation [2]. Deep learning (DL) has recently been widely employed for high-performance image-analysis tasks such as object recognition [3,4,5], image segmentation [2,6,7,8,9], and image classification [1,10,11,12]. The ability to distinguish objects and properties in images (for example, cancer cells in biopsy samples) is changing the way clinical samples are evaluated. In this study, we present a soft label fully convolutional network (SL-FCN) for automatic segmentation of human epidermal growth factor receptor 2 (HER2) amplification in fluorescence in situ hybridization (FISH) and dual in situ hybridization (DISH) images of invasive breast cancer and papillary thyroid carcinoma (PTC) on Papanicolaou-stained FNA and thin prep (TP) whole slide images (WSIs).

**Figure 1:**
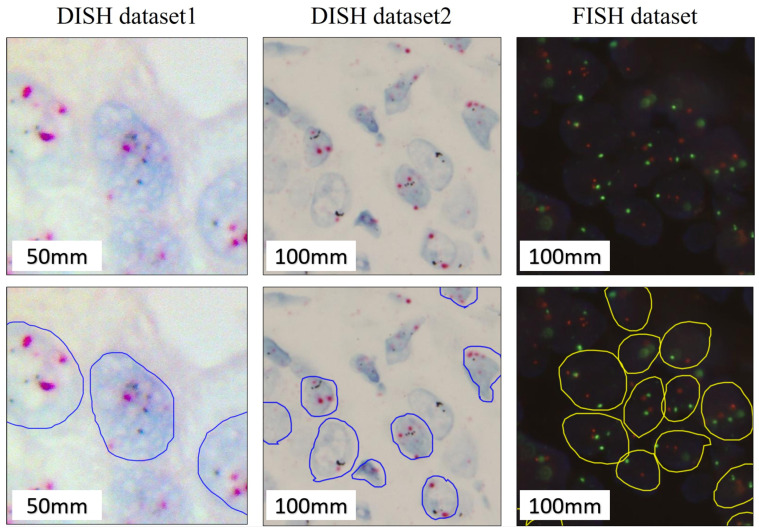
An illustration of DISH and FISH images including unclear and blurry cell boundaries and large variations on cell shapes and signals. (**up**) a partial view of a DISH image with (**bottom**) the annotations by the pathologists.

Breast cancer remains the most frequently diagnosed cancer and the leading cause of cancer death among females worldwide [13]. Human epidermal growth factor receptor 2 gene amplification (HER2; ERBB2) test is well established to determine whether a breast cancer patient is eligible for anti-HER2 target therapy [14,15]. When breast cancer treated with anti-HER2 target therapies, such as trastuzumab, pertuzumab, and tyrosine kinase inhibitor lapatinib and neratinib, they have been shown to significantly improve survival, but without appropriate anti-HER2 therapy, HER2-amplified tumors are associated with poor prognosis [16,17,18,19,20,21,22]. Although immunohistochemistry (IHC) is a good screening method for negative (0+ or 1+) and strong positive (3+) results, any patient with IHC equivocal positive result (2+) should be confirmed by fluorescence in situ hybridization (FISH) analysis for anti-HER2 target therapies [23]. Dual in situ hybridization (DISH) can be used for signal visualization and the benefit of simultaneous morphologic correlation using light microscopy, and there is no need for specialized fluorescence equipment [24,25]. FISH and DISH both use dual probes to highlight the HER2 gene and the chromosome 17 centromere (CEN17) in different colors. The main distinction between positive and negative amplification status is based on the HER2/CEN17 ratio and the average HER2 copy number per nucleus in at least 20 nuclei. The American Society of Clinical Oncology (ASCO)/College of American Pathologists (CAP) initially issued a detailed guideline for clinical testing and interpretation of HER2 results in 2007, which were first revised in 2013 and updated in 2018. Based on the 2018 ASCO–CAP guidelines, the result is classified into five categories by FISH; group 1: When the HER2/CEN17 ratio is ≥2.0, and the average HER2 gene copy number ≥ 4 is reported as positive; group 2: When the HER2/CEN17 ratio is ≥2.0, and HER2 gene copy number < 4 is reported as negative, unless concurrent IHC 3+; group 3: When HER2/CEN17 ratio is <2.0, and HER2 gene copy number ≥ 6 is reported as negative, unless concurrent IHC 2+ or 3+; group 4: When HER2/CEN17 ratio is <2.0, and HER2 gene copy number ≥ 4 and <6 is reported as negative, unless concurrent IHC 3+; group 5: When HER2/CEN17 ratio is <2.0, and HER2 gene copy number < 4 is reported as negative [24,26]. Accurate assessment of HER2 status is an essential step to identify the subset of breast cancer patients who may benefit from the anti-HER2 targeted therapy [17,26,27,28]. Manual assessment of the HER2 amplification status is very time-consuming, laborious, and error-prone. The automated medical images diagnostic method is arguably the most successful field of medical applications that can dramatically increase the time efficiency for the pathologist’s analysis and improve the accuracy of counting [29,30,31]. The development of image analysis based on new artificial intelligence (AI)-based approaches in pathology is being led by computer engineers and data scientists can also be used to improve diagnostic accuracy for clinical precision decision-making in cancer treatment [31]. However, analysis of HER2 expression is challenging due to unclear and blurry cell boundaries with large variations on cell shapes and signals as illustrated in Figure 1.

Our research is the first attempt to use soft label FCN technology for automatic segmentation of HER2 amplification in FISH and DISH images of invasive breast cancer. In evaluation, to test the model robustness and model generalizability, three clinical datasets were collected using different magnifications from the Tri-service general hospital in Taipei, Taiwan. The pathologists produced a reference standard by manually annotating the HER2, ERBB2, and CEN17 signals in the FISH and DISH images. We compare the proposed algorithms with thirteen popular or recently published deep learning methods, including U-Net [2] +InceptionV4 [32], Ensemble of U-net with Inception-v4 [32], Inception-Resnet-v2 encoder [32], and ResNet-34 encoder [33], SegNet [34], Modified FCN [6,7,8,9,10,11], YOLOv5 [35], FCN [36], CPN [37], SOLOv2 [38], BCNet [39], and Deeplabv3+ [40] with three different backbones, including MobileNet [41], ResNet [33], and Xception [42] (see Section 4). The algorithms we developed are more objective, precise, and unbiased than the current standard manual interpretation results for anti-HER2 target therapy.

Thyroid cancer has one of the highest occurrences among the numerous forms of cancer [43]. The most frequent kind of thyroid cancer is papillary thyroid carcinoma (PTC). The study of a fine needle aspiration biopsy (FNAB), which is stained and spread onto a glass slide, is the most essential test in the preliminary detection of thyroid cancer [44]. A cytopathologist examines the FNAB sample under an optical microscope to estimate the risk of malignancy based on numerous aspects of thyroid cells, such as size, color, and cell group architecture. Digital pathology has just recently developed as a potential new standard of treatment in which glass slides are transformed into whole slide images (WSIs) utilizing digital slide scanners. Due to the very large size of a typical WSI (on the order of gigapixels), pathologists consider it challenging to manually detect all the information in WSI. Thus, artificial intelligence-based automated diagnosis approaches are being explored to solve the restrictions of manual and complicated diagnosis processes. In this study, we develop a soft labeled FCN based deep learning framework for the automatic segmentation of PTC in WSIs. To evaluate the robustness and generalizability of the proposed method, the clinical dataset containing 131 Papanicolaou-stained WSIs was collected from Tri-Service general hospital in Taipei, Taiwan. The reference standard was manually generated by annotating tumor cells in Papanicolaou-stained WSIs. In evaluation, the proposed method is compared with three state-of-the-art deep learning methods, including Modified FCN [6,7,8,9,10,11], U-Net [2], and SegNet [34].

## 2. Related Works in Soft Label, Label Smoothing, and Segmentation Approaches

In this section, we discuss three categories of works, which are most related to our proposed method, including soft label techniques, label smoothing methods, and segmentation approaches.

### 2.1. Soft Label Techniques

In traditional segmentation methods, the network usually receives binary ground truth labels or hard labels (label values are 0 and 1 only), which may cause information loss, especially for the pixels at the boundary between two different types [45]. To prevent this limitation, instead of hard labels, researchers [45,46,47] propose to use soft labels (label values are continuous values between 0 and 1), which can preserve more image information throughout the training process [47]. Soft label approaches have improved generalization, accelerated learning, and reduced network over-confidence [45,46,47]. When computing segmentation-based morphometric measurements, SoftSeg, a method based on U-Net [48] architecture proposed by Gros et al. [45], makes better precision than traditional binary segmentations (increase in 6.5% of DICE on the 2019 BraTS dataset) and has increased sensitivity, which is desired by radiologists. Zhang et al. [49] compared the segmentation result between using hard labels and soft labels and demonstrated that using soft labels can increase the segmentation performance. Engelen et al. [50] proposed to blur the ground truth mask with a Gaussian filter for label softening and demonstrated the improvement in in-vivo MRI and CT angiography (CTA) [51] images dataset. Qi et al. [52] developed a novel Progressive Cross-camera Soft-label Learning (PCSL) framework for the semi-supervised person re-identification task that enhanced feature representations through a different learning method. Kats et al. [47] proposed a modified simultaneous truth and performance level estimation (STAPLE) [53] algorithm for soft annotations of experts and demonstrated that training the fully convolution neural network with the soft labels improves generalization and performance gain.

### 2.2. Label Smoothing Methods

It is widely known that neural network training is sensitive to the loss that is minimized [46]. Instead of using hard labels for model training, labeling smoothing methods utilize soft labels that are generated by exploiting a uniform distribution to smooth the distribution of the hard labels and aim at providing regularization for a learnable classification model [49]. Label smoothing is a method commonly used in training deep learning models to keep the neural network from becoming over-confident and to enhance model calibration and segmentation performance [46]. The label smoothing approach has been utilized in the fields of medical image analysis [54,55], style transfer [56], speech recognition [57], and language translation [58] to improve the performance of the deep learning models. For example, Müller et al. [46] demonstrated that label smoothing implicitly calibrates learned models so that the confidences of their predictions are more aligned with the accuracies of their predictions. Li et al. [54] developed a ground truth softening methodology using the over-segmentation algorithm and smoothing based on the distance to an annotated boundary, and the experimental results demonstrate that using soft labels improves the model performance on both 2D and 3D medical images (increase in 0.7% of Dice on the MRBrainS18 dataset [59]). Zhao et al. [56] proposed an approach, which automatically segments items and extracts their soft semantic masks from the style and content images, to preserve the structure of the content image while having the style transferred. Pham et al. [55] developed a labeling smoothing method to better handle uncertain samples, which constitute a significant portion of chest X-ray datasets. Zhang et al. [49] presented an Online Label Smoothing (OLS) strategy, which generates soft labels based on the statistics of the model prediction for the target category, and demonstrates that the performance of the OLS method is better than other regularization approaches on the Canadian Institute for Advanced Research-100 (CIFAR-100) dataset [60].

### 2.3. Segmentation Approaches

Segmentation models are widely used in automated medical image analysis and have shown good performance [6,36,38,40]. A fully convolutional network (FCN) is introduced by Shelhamer et al. [36] for semantic image segmentation. To produce accurate and detailed segmentations, they defined a skip architecture that combines semantic information from a deep, coarse layer with appearance information from a shallow, fine layer. In recent years, researchers developed a modified FCN-32s approach and demonstrated that it is beneficial for tumor segmentation in the diagnosis of cervical cancer [7], thyroid cancer [6], breast cancer [8], ovarian cancer [10,11], and EBUS [9]. Shen et al. [61] developed a modified mini-U-net to segment the touching cells accurately in FISH images and demonstrated that the performance is better than the original mini-U-net [62]. Upschulte et al. [37] built a Contour Proposal Networks (CPNs), a framework for object instance segmentation by proposing contours that are encoded as fix-sized representations based on Fourier Descriptors, and evaluated the performance on three datasets (NCB, BBBC039 [63], SYNTH), which contains the large variations in cell shapes. Ke et al. [39] proposed a Bilayer Convolutional Network (BCNet), a bilayer mask prediction network for addressing the issues of heavy occlusion and overlapping objects in two-stage instance segmentation, and evaluated the performance on the COCO dataset [64]. Wang et al. [38] designed a dynamic instance segmentation framework called Segmenting Objects by Locations v2 (SOLOv2) and showed its robustness using the MS COCO dataset [64], which includes 91 stuff categories of per-pixel segmentation masks. Chen et al. [40] proposed DeepLabv3+, a deep learning model with an encoder–decoder structure, and proved its efficacy on the Cityscapes dataset [65], which includes polygonal annotations of instance segmentation for vehicles and people. In our experiment, we compare the proposed method with the state-of-the-art deep learning models, including FCN [36], Modified FCN [6,7,8,9,10,11], U-Net [2] +InceptionV4 [32], Ensemble of U-Net with Inception-v4 [32], Inception-Resnet-v2 encoder [32], and ResNet-34 encoder [33], U-Net [2], SegNet [34], YOLOv5 [35], BCNet [39], CPN [37], SOLOv2 [38], and DeepLabv3+ [40] with three different backbones, including MobileNet [41], ResNet [33], and Xception [42].

## 3. Materials and Methods

### 3.1. Materials

The performance of the proposed deep learning model is evaluated using four datasets, including two DISH breast datasets obtained on two different magnification levels, a FISH breast dataset, and a Papanicolaou-stained FNA and TP thyroid dataset. Ethical approvals have been obtained from the research ethics committee of the Tri-Service General Hospital (TSGHIRB No.1-107-05-171 and No.B202005070), and the data were de-identified and used for a retrospective study without impacting patient care. For FISH and DISH images of invasive breast cancer, we select patients coming to our medical center for breast cancer treatment who had infiltrating ductal carcinoma pathology diagnoses. De-identified, digitized images of Dual-color FISH and DISH in HER2 IHC scores 2+ equivocal cases from January 2014 to December 2021 were obtained from the tissue bank of the Department of Pathology, Tri-Service General Hospital, National Defense Medical Center, Taipei, Taiwan (*n* = 470, including 200 FISH images and 270 DISH images with two different device magnifications). For the DISH breast dataset 1, the slides were collected with 1200× overall magnification using 20× eyepiece lens (Forever Plus Corp., Taiwan) and 60× objective lens (Olympus, Japan). For the DISH breast dataset 2 and FISH breast dataset, the slides were collected with 600× overall magnification using 10× eyepiece lens (Olympus, Japan) and 60× objective lens (Olympus, Japan). DISH and FISH results were evaluated independently by two pathologists, generating annotations of invasive breast cancer areas of each slide to highlight individual tumor cells with associated labels for HER2 and CEN17 signals. For Papanicolaou-stained FNA and TP cytological slides for thyroid cancer diagnosis, de-identified and digitized 131 WSIs were received from the Department of Pathology, Tri-Service General Hospital, Taipei, Taiwan, comprising 120 PTC cytologic slides (smear, Papanicolaou-stained, *n* = 120) and 11 PTC cytologic slides (TP, Papanicolaou-stained, *n* = 11). Table 1 presents the detailed information of experimental datasets.

#### 3.1.1. Fish Breast Dataset

The PathVysion HER2 DNA probe kit II (Vysis Inc., Downers Grove, IL, USA) was performed following the manufacturer’s instructions, which is designed to detect amplification of the HER2 gene via FISH in formalin-fixed paraffin-embedded (FEPE) human breast cancer tissue specimens. FISH is performed using a dual probe highlighting the HER2 gene and the CEN17 in a different color. The FFPE tissue blocks containing breast cancer were selected and regions of interest were marked on hematoxylin and eosin (H and E) slides. The selected area in the subsequent section was taken for FISH analysis. Tissues were subjected to a series of deparaffinization, dehydration, and prehybridization treatments. After this time, probes were added, and the sections were left to incubate overnight. After post-hybridization washes, sections were mounted and checked for signal. The entire slide was screened, and every single discrete nucleus was examined for red and green signals.

#### 3.1.2. Dish Breast Datasets

This study is performed by using the INFORM HER2 Dual ISH DNA Probe Cocktail Assay from Ventana Medical Systems, which is a dual-color DISH assay. The HER2 gene is detected by a dinitrophenyl (DNP)-labeled probe and visualized using an ultraView silver in situ hybridization (SISH) DNP detection Kit. The CEN17 is targeted using a digoxigenin (DIG)-labeled probe and detected using an ultraView Red ISH DIG detection Kit. Under light microscopy, HER2 shows as discrete black signals, and chromosome 17 appears as red signals. The sections were loaded into the Ventana Benchmark XT machine. A fully automated procedure was carried out with the following basic steps: Deparaffinization, followed by cell conditioning, and protease digestion. Following that, the probe was applied followed by hybridization and application of the SISH Multimer. Following that, the silver chromogen was applied and then followed by the application of Red ISH Multimer and red chromogen. Finally, hematoxylin was used to counterstain the image, which was followed by clearing in xylene and mounting with dibutyl phthalate polystyrene xylene.

#### 3.1.3. FNA and TP Thyroid Dataset

The screening of cytology slides was first performed by cytologists, and two experienced pathologists confirmed these papillary carcinoma tumor groups labeled by cytologists. Cytology was performed using a 2017 Bethesda System for reporting thyroid cytopathology. The well-preserved thyroid FNAs performed during the previous two years are chosen. All stained slides were scanned at 20× objective magnification with a Leica AT Turbo (Leica, Germany) and the average slide size is 77,338 × 37,285 pixels. Two experienced pathologists created the reference standard. The training model uses a total of 28 Papanicolaou-stained WSIs (21%), including 25 thyroid FNA and three TP cytologic slides. The remaining 103 Papanicolaou-stained WSIs (79%), including 95 thyroid FNA and eight TP cytologic slides, are used as a separate testing set for evaluation.

### 3.2. Proposed Method: Soft Label FCN

A fully convolutional network (FCN) is introduced by Shelhamer et al. [36] for semantic image segmentation, and the proposed method is an extended improved model of our previous effort, i.e., a modified FCN, which has been demonstrated to be highly effective for tumor segmentation in the diagnosis of thyroid cancer [6], cervical cancer [7], breast cancer [8], ovarian cancer [10,11], and EBUS [9] and showed better segmentation performance than the original FCN [36] and a number of popular deep learning approaches. However, when dealing with objects of interest with blurry or unclear boundaries, the performance of existing deep learning models declines as shown in our experiment. To deal with this issue, we propose an improved soft-labeled FCN architecture to achieve better results, especially for data with blurry or unclear cell borders for semantic image segmentation. By utilizing soft labels instead of hard labels, the image information loss during the training process could be reduced [47]. Recent studies show that label smoothing can improve the segmentation performance at the boundaries of different regions [54,55,56]. In our study, we proposed a new loss function, namely the soft weight softmax loss function, which utilizes soft labels and integrates the concept of a label smoothing method [45,54] into the softmax loss function (see Section 3.2.1 and Section 3.2.2) to improve the image segmentation results on data with blurry or unclear cell boundaries.

**Figure 2:**
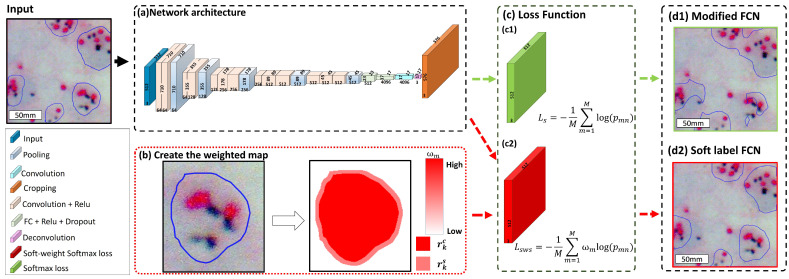
The main architecture of the soft-labeled FCN in DISH breast dataset 1. (**a**) the soft-labeled FCN network architecture; (**b**) create the weighted map and obtain the pixel weight $\omega_{m}$ as the input for soft-weight softmax loss; (**c**) the loss function comparison; (**c1**) is the original loss function in the modified FCN network; (**c2**) is the soft-weight softmax loss in our proposed method; (**d1**) is the output result of the modified FCN network; (**d2**) is the output result of the soft-labeled FCN network.

The major modification of the proposed soft-labeled FCN is the replacement of the original softmax loss function with a new soft weight softmax loss function, which assigns lower weights to the blurry and unclear cell bordering regions and higher weights to the center regions of annotations in computing the model loss. This helps build models focusing on the center annotated regions of interest with higher confidence (by assigning higher weight), and in the meantime, for confusing bordering regions, with lower attention in these blurry or unclear cell borders while training. Figure 2 presents the workflow of the proposed framework.

#### 3.2.1. Soft Label Modeling

The efficacy of using soft labels instead of hard labels has been demonstrated in many research [45,46,47]. To improve the performance of boundary segmentation, we devise a soft label modeling for training better models. We convert these annotations *A* into bounding boxes $B={\left\{b_{k}\right\}}_{k=1,2,...K}$ which could be formulated as follows:

(1) $$b_{k}=\left(\min\left(i_{r_{k}^{a}}\right),\min\left(j_{r_{k}^{a}}\right),w_{b_{k}},h_{b_{k}}\right)$$

(2) $$w_{b_{k}}=\max\left(i_{r_{k}^{a}}\right)-\min\left(i_{r_{k}^{a}}\right)$$

(3) $$h_{b_{k}}=\max j_{r_{k}^{a}}-\min j_{r_{k}^{a}}$$

where $i_{r_{k}^{a}}$ represents the *x*-axis coordinate of the *k*-th annotation, $j_{r_{k}^{a}}$ represents the *y*-axis coordinate of the *k*-th annotation, $w_{b_{k}}$ denotes the width of the *k*-th bounding box, and $h_{b_{k}}$ denotes the height of the *k*-th bounding box.

We define $\psi ={\left\{\psi_{k}\right\}}_{k=1,2,...K}$ as a set of the diagonal lines of bounding box in the training dataset, and the diagonal line $\psi_{k}$ can be formulated as follows:

(4) $$\psi_{k}=\sqrt{{w_{b_{k}}}^{2}+{h_{b_{k}}}^{2}}$$

After the ψ has been generated, we arrange the elements of ψ in an ascending order, and let $\psi^{\prime }$ denote the set of diagonal lines after sorting which is formulated as follows:

(5) $$\psi^{\prime }=\left\{\psi_{1}^{\prime },\psi_{2}^{\prime },...\psi_{K}^{\prime }\right\},\psi_{1}^{\prime }\leq \psi_{2}^{\prime }\leq ...\leq \psi_{K}^{\prime }$$

The median of diagonal line $\psi^{*}$ is calculated as follows:

(6) $$\psi^{*}=\left\{\begin{array}{cc} \psi_{\frac{\left(K+1\right)}{2}}^{\prime } & ,K\%2=1\\ \frac{1}{2}\left(\psi_{\frac{K}{2}}^{\prime }+\psi_{\frac{K}{2}+1}^{\prime }\right) & ,otherwise \end{array}\right.$$

where % represents the remainder operator.

Given $\psi^{*}$, the erosion kernel size $\kappa^{e}$ and dilation kernel size $\kappa^{d}$ could be formulated as follows:

(7) $$\kappa^{e}=\upsilon \left\lfloor \phi \psi^{*}+\frac{1}{2}\right\rfloor +1$$

(8) $$\kappa^{d}=\tau \left\lfloor \phi \psi^{*}+\frac{1}{2}\right\rfloor +1$$

where ϕ, υ and τ are empirically determined to scale the kernel size; ϕ = 0.01, υ = 2, and τ = 6.

Given $F[\kappa^{e}]$ and $F[\kappa^{d}]$ representing two binary structuring elements, each with a morphological kernel size ($\kappa^{e}$ and $\kappa^{d}$) for erosion and dilation operations, the $F[\kappa^{e}]$ and $F[\kappa^{d}]$ could be formulated as follows:

(9) $$F\left[\kappa^{e}\right]={\left[\begin{array}{ccc} 1 & \cdots & 1\\ \vdots & \ddots & \vdots \\ 1 & \cdots & 1 \end{array}\right]}_{\kappa^{e}\times \kappa^{e}}$$

(10) $$F\left[\kappa^{d}\right]={\left[\begin{array}{ccc} 1 & \cdots & 1\\ \vdots & \ddots & \vdots \\ 1 & \cdots & 1 \end{array}\right]}_{\kappa^{d}\times \kappa^{d}}$$

Let $R^{c}={\left\{r_{k}^{c}\right\}}_{k=1,2,...,K}$ denote the $r_{k}^{a}$ region after erosion operation, which is formulated by Equation (11), and $R^{o}={\left\{r_{k}^{o}\right\}}_{k=1,2,...,K}$ denotes the $r_{k}^{c}$ region after dilation operation, which is calculated with Equation (12):

(11) $$r_{k}^{c}=r_{k}^{a}⊖F[\kappa^{e}]$$

(12) $$r_{k}^{o}=r_{k}^{c}⊕F[\kappa^{d}]$$

where ⊕ and ⊖ denote the binary morphological dilation and erosion operations.

Given $R^{c}$ and $R^{o}$, the erosion region $R^{e}={\left\{r_{k}^{e}\right\}}_{k=1,2,...K}$ and the dilation region $R^{d}\;=\;{\left\{r_{k}^{d}\right\}}_{k=1,2,...K}$ could be formulated as follows:

(13) $$r_{k}^{e}=r_{k}^{a}\cap \left(∼r_{k}^{c}\right)$$

(14) $$r_{k}^{d}=r_{k}^{o}\cap \left(∼r_{k}^{a}\right)$$

However, on the other hand, the soft label regions $R^{s}={\left\{r_{k}^{s}\right\}}_{k=1,2,...K}$ are the union of erosion regions and dilation regions, which is formulated as follows:

(15) $$r_{k}^{s}=r_{k}^{e}\cup r_{k}^{d}$$

After generating soft label map, we model the loss weight $\omega_{\left(m\right)}$ of each pixel at *m* as formulated in Equation (16):

(16) $$\omega_{(}m)=\left\{\begin{array}{cc} \Psi & ,m\in R^{c}\\ \Pi & ,m\in R^{s}\\ ℵ & ,otherwise \end{array}\right.$$

where Ψ, Π, and *ℵ* are empirically determined; Ψ=2, Π=1.5, and ℵ=1.

As shown in Equation (16), the higher weights are assigned to the center of annotation $R^{c}$ so that the model can focus on these regions during the training process while assigning lower weights to the boundary regions, which include blurry or unclear cell boundaries ($R^{s}$) and lowest weights to the background to reduce their influence on gradients during the training process.

#### 3.2.2. Soft Weight Softmax Loss Function

The softmax loss function is popular in image segmentation models [6,7,34,36,56]. Based on the original softmax loss function, we proposed to utilize a new loss function that can preserve more image information and reduce the influences caused by the confusing regions during the training process. In this paper, we built a soft weight softmax loss function $L_{sws}$ to help the model focus on the central regions of interest with high confidence while reducing the attention on blurry or unclear cell borders.

Shown as Figure 2(c1), the original softmax loss function $L_{s}$ in modified FCN architecture [6,7,8,9,10,11] could be formulated as follows:

(17) $$L_{s}=-\frac{1}{M}\sum_{m=1}^{M}\log\left(p_{mn}\right)$$

where *M* is the number of pixels of training data, and $p_{mn}$ is formulated as follows:

(18) $$p_{mn}=\frac{e^{z_{mn}}}{\sum_{t=1}^{N}e^{z_{mt}}}$$

where *N* denotes the number of classes, $z_{mn}$ is the predicted score *z* for pixel *m* belonging to the target class *n*; and $z_{mt}$ denotes the predicted score *z* belonging to *t*-th class (t ∈ [1,N]) in pixel *m*.

Figure 2(c2) shows the soft weight softmax loss function $L_{sws}$ in our proposed soft label FCN, which is formulated by adding the soft weight. The soft weight softmax loss function is formulated as follows:

(19) $$L_{sws}=-\frac{1}{M}\sum_{m=1}^{M}\omega_{m}\log\left(p_{mn}\right)$$

where $\omega_{m}$ is the weight value ω belonging to the pixel *m*. The center of annotations $R^{c}$ has been assigned the highest weights in computing model loss so that the model can focus on training the central regions with high confidence. On the other hand, the boundary regions which include erosion regions $R^{E}$, dilation regions $R^{D}$, and the background regions have been assigned lower weights in computing model loss to reduce the confusion caused by these regions while training. By assigning these regions with different weights in the loss function, the model can focus on the target regions and reduce the confusion by other regions.

#### 3.2.3. Proposed Soft-Labeled FCN Architecture

Based on the modified FCN [6,7,8,9,10,11], we proposed a soft-labeled FCN that is improved from the FCN-32 architecture, which is shown in Figure 2a. Firstly, the network requires 512×512 tiles as an input image. The first two stages consist of two convolutional layers with a filter size of 3×3, a stride of 1, and the ReLU, then the max-pooling layer with 2×2 filter size and stride of 2 comes next to the convolutional layer. The next three stages consist of three convolutional layers with the filter size of 3×3, the stride of 1 and the ReLU comes next to the convolutional layer, the max-pooling layer with the filter size of 2×2 and the stride of 1 is followed by the convolutional layer. After three convolutional layers, the next two stages consist of a fully connected (FC) layer with 3×3 filter size, stride 1, ReLU, and dropout layer. Next, the convolutional layer with 1×1 kernel size, and then the deconvolutional layer with kernel size 64×64 and stride of 32 is utilized to upsample the feature maps. After the deconvolutional layer is the cropping layer. Following cropping, the last layer of the model is the loss function. Figure 2b demonstrates the process of obtaining weight value in the proposed loss function using the soft label modeling (see Section 3.2.1). The detailed information about the proposed soft weight softmax loss function and its comparison with the softmax loss function is described in Section 3.2.2. Figure 2d presents the output segmentation results from the traditional softmax loss function (Figure 2(d1)) and the proposed loss function (Figure 2(d2)). It can be seen that the proposed loss function has improved the performance of the model. The detailed framework of the proposed soft label FCN is presented in Figure 2. The detailed architecture of the proposed deep learning network is shown in Table 2.

#### 3.2.4. Implementation Details

To train the proposed method, the model is initialized by the VGG16 model, optimized with the SGD optimizer, and using the soft weight loss as the loss function. Moreover, the base learning rate in the proposed method is $1\times 10^{-10}$, weight decay of $5\times 10^{-4}$, and momentum of 0.99. Data augmentation is also utilized as a regularizer in neural networks, minimizing overfitting and improving performance when dealing with unbalanced classes. For data argumentation, we rotate our input images per $5^{\circ }$ and 5 times, increment of $90^{\circ }$, and flip our input images along the horizontal and vertical axes during the training process.

## 4. Results

### 4.1. Evaluation Metrics

For quantitative evaluation, we utilize the accuracy, precision, recall, F1-score, and Jaccard index to compare and measure the performance of the benchmark approaches and the proposed method. The metrics are calculated as follows:

(20) $$Accuracy=\frac{TP+TN}{TP+TN+FP+FN}$$

(21) $$Precision=\frac{TP}{TP+FP}$$

(22) $$Recall=\frac{TP}{TP+FN}$$

(23) $$F1\;score=\frac{2TP}{2TP+FP+FN}$$

(24) $$Jaccard\;index=\frac{TP}{TP+FP+FN}$$

where *TP* represents the true positive, *TN* is the true negative, *FP* denotes false positive, and *FN* is the false negative.

### 4.2. Quantitative Evaluation with Statistical Analysis in DISH Breast Dataset 1

**Figure 3:**
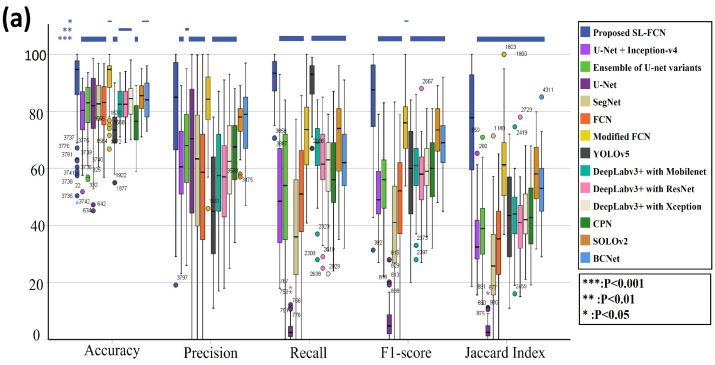

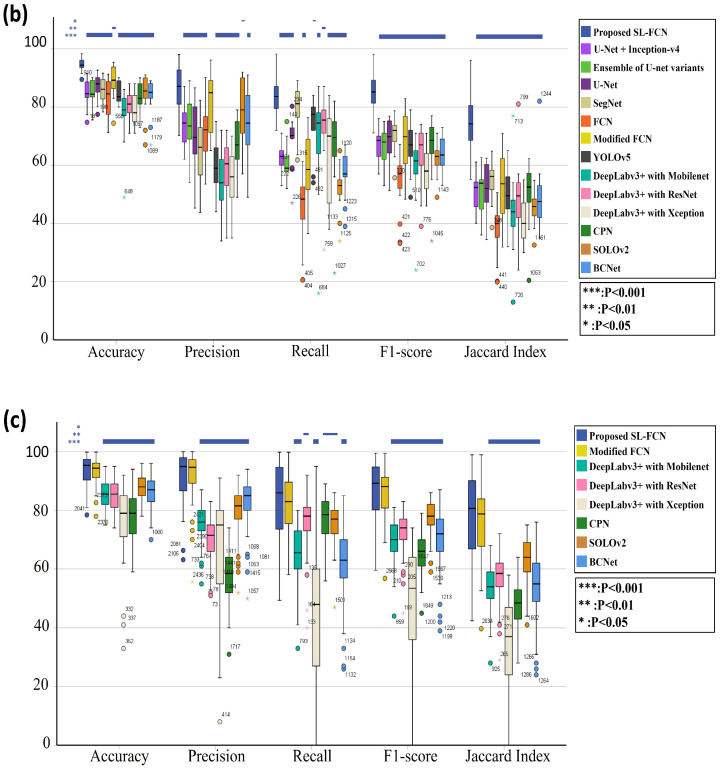
The box plots of quantitative evaluation results of the three breast cancer datasets, including (**a**) DISH dataset 1; (**b**) DISH dataset 2; (**c**) FISH dataset.

**Figure 4:**
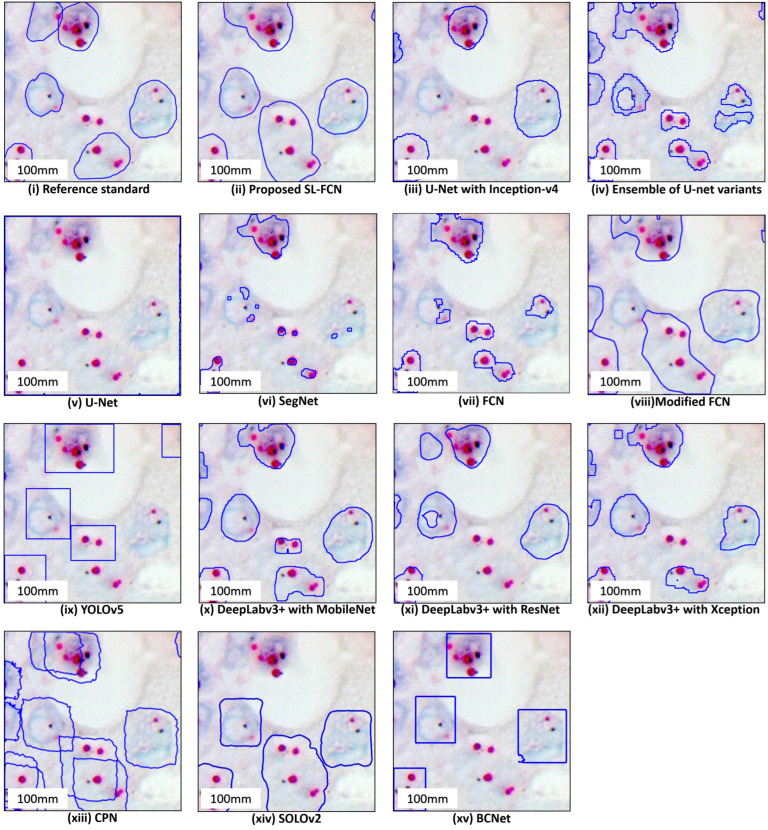
Qualitative segmentation results of the proposed SL-FCN method and the baseline methods for segmentation of HER2 amplification in DISH dataset 1.

The quantitative evaluation results in segmentation of HER2 amplification in DISH dataset 1 are presented in Table 3a. The proposed Soft-label FCN in segmentation of HER2 amplification of DISH dataset 1 with an accuracy of 87.77 ± 14.97%, precision of 77.19 ± 23.41%, recall of 91.20 ± 7.72%, F1-score of 81.67 ± 17.76%, and Jaccard Index of 72.40 ± 23.05%. In addition, the box plots of the quantitative assessment results for breast cancer segmentation are shown in Figure 3a, demonstrating that the suggested technique consistently outperforms the baseline approaches. To further demonstrate the efficacy and efficiency of the proposed method, using SPSS software, we examined that the quantitative scores were evaluated with Fisher’s Least Significant Difference (LSD) (Table 4). Based on the LSD test, the suggested approach substantially exceeds most of the baseline approaches in terms of precision, recall, F1-score, and Jaccard index (*p* < 0.001). Figure 4 presents the visual comparison of segmentation results of the proposed method and the baseline approaches for segmentation of HER2 amplification. Here, we can observe a consistency between the typical segmentation results generated by the proposed method and the reference standard produced by an expert pathologist. Results from the quantitative and qualitative evaluation show that the proposed soft label FCN outperforms the baseline models, including U-Net [2] with InceptionV4 [32], Ensemble of U-net with Inception-v4 [32], Inception-Resnet-v2 encoder [32], and ResNet-34 encoder [33], SegNet [34], Modified FCN [6,7,8,9,10,11], U-Net [2], YOLOv5 [35], FCN [36], CPN [37], SOLOv2 [38], BCNet [39], and Deeplabv3+ [40] with three different backbones, including MobileNet [41], ResNet [33], and Xception [42].

### 4.3. Quantitative Evaluation with Statistical Analysis in DISH Breast Dataset 2

**Figure 5:**
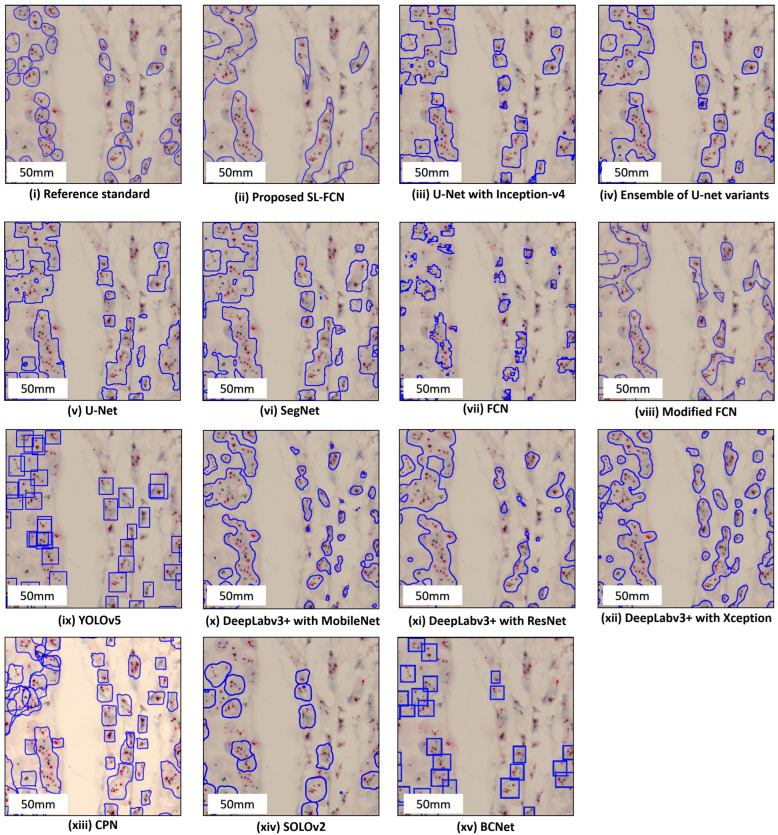
Qualitative segmentation results of the proposed SL-FCN method and the baseline methods for segmentation of HER2 amplification in DISH dataset 2.

The quantitative evaluation results in the segmentation of HER2 amplification in DISH dataset 2 are presented in Table 3b. The proposed soft label FCN in segmentation of HER2 amplification of DISH dataset 2 with an accuracy of 94.64 ± 2.23%, precision of 86.78 ± 1.07%, recall of 83.78 ± 6.42%, F1-score of 85.14 ± 6.61%, and Jaccard Index of 74.67 ± 10.05%. In addition, the box plots of the quantitative assessment results for breast cancer segmentation are shown in Figure 3b, demonstrating that the suggested technique consistently outperforms the baseline approaches. To further demonstrate the efficacy and efficiency of the proposed method, using SPSS software, we examined the quantitative scores that were evaluated with Fisher’s Least Significant Difference (LSD) (Table 5). Based on the LSD test, the suggested approach substantially exceeds the baseline approaches in terms of precision, recall, F1-score, and Jaccard index (*p* < 0.001). Figure 5 presents the visual comparison of segmentation results of the proposed method and the baseline approaches for segmentation of HER2 amplification. Here, we can observe a consistency between the typical segmentation results generated by the proposed method and the reference standard produced by an expert pathologist. Results from the quantitative and qualitative evaluation show that the proposed soft label FCN outperforms the baseline models, including U-Net [2] with InceptionV4 [32], Ensemble of U-Net with Inception-v4 [32], Inception-Resnet-v2 encoder [32], and ResNet-34 encoder [33], SegNet [34], Modified FCN [6,7,8,9,10,11], U-Net [2], YOLOv5 [35], FCN [36], CPN [37], SOLOv2 [38], BCNet [39], and Deeplabv3+ [40] with three different backbones, including MobileNet [41], ResNet [33], and Xception [42].

### 4.4. Quantitative Evaluation with Statistical Analysis in the FISH Breast Dataset

**Figure 6:**
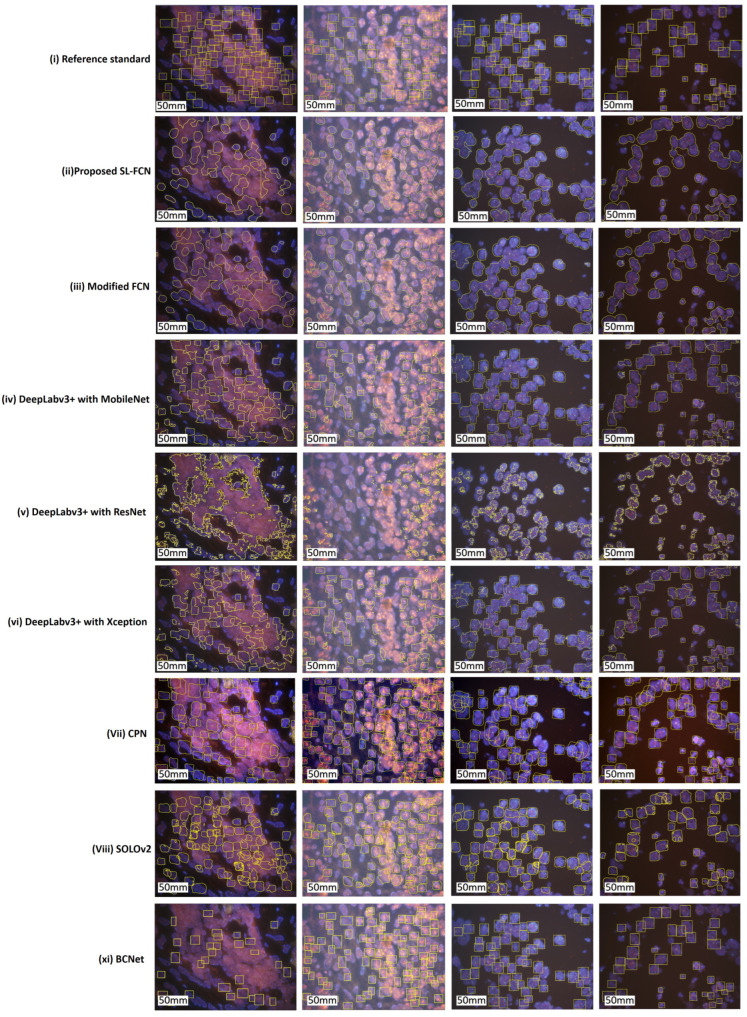
Qualitative segmentation results of the proposed SL-FCN method and the baseline methods for segmentation of HER2 amplification in the FISH dataset.

The quantitative evaluation results in the segmentation of HER2 amplification in FISH dataset are presented in Table 3c. The proposed soft label FCN for HER2 amplification of FISH dataset with an accuracy of 93.54 ± 5.24%, precision of 91.75 ± 8.27%, recall of 83.52 ± 13.15%, F1-score of 86.98 ± 9.85%, and Jaccard Index of 78.22 ± 14.73%. In addition, the box plots of the quantitative assessment results for breast cancer segmentation are shown in Figure 3c, demonstrating that the suggested technique consistently outperforms the baseline approaches. To further demonstrate the efficacy and efficiency of the proposed method, using SPSS software, we examined the quantitative scores that were evaluated with Fisher’s Least Significant Difference (LSD) (Table 6). Based on the LSD test, the suggested approach substantially exceeds the baseline approaches in terms of precision, recall, F1-score, and Jaccard index (*p* < 0.001). Figure 6 presents the visual comparison of segmentation results of the proposed method and the baseline approaches for segmentation of HER2 amplification. Here, we can observe a consistency between the typical segmentation results generated by the proposed method and the reference standard produced by an expert pathologist. Results from the quantitative and qualitative evaluation show that the proposed soft label FCN outperforms the baseline models, including Modified FCN [6,7,8,9,10,11], YOLOv5 [35], CPN [37], SOLOv2 [38], BCNet [39], and Deeplabv3+ [40] with three different backbones, including MobileNet [41], ResNet [33], and Xception [42].

### 4.5. Quantitative Evaluation with Statistical Analysis in the Thyroid Dataset

**Figure 7:**
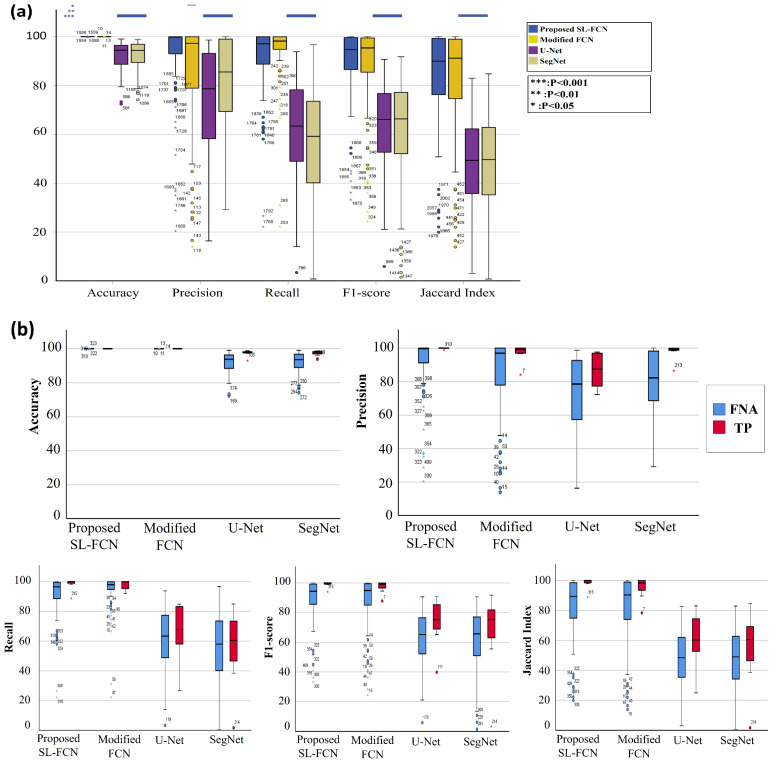
The box plots of quantitative evaluation results of the thyroid cancer dataset, including (**a**) overall thyroid cancer dataset and (**b**) the thyroid FNA and TP cytological slides.

**Figure 8:**
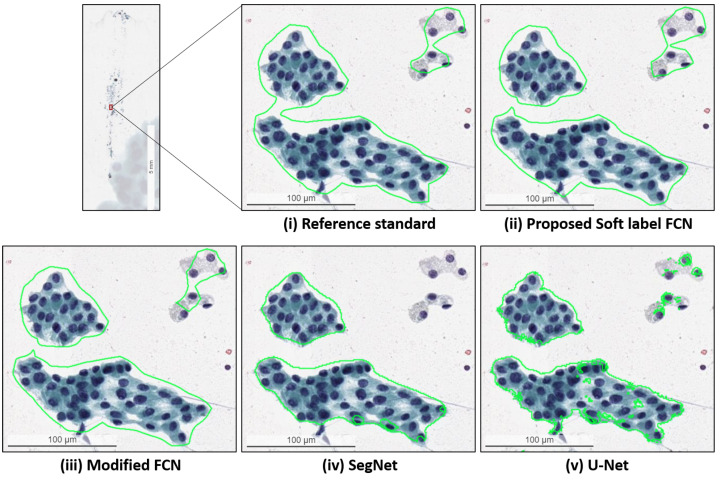
Qualitative segmentation results of the proposed SL-FCN method and the baseline methods for segmentation of PTC in Papanicolaou-stained WSIs.

The quantitative evaluation results for the segmentation of PTC in Papanicolaou-stained FNA and TP WSIs are presented in Table 7a. The experimental results demonstrate that the proposed SL-FCN achieves superior performance compared to the baseline approaches, including Modified FCN [6,7,8,9,10,11], U-Net [2], and SegNet [34] with an accuracy of 99.99 ± 0.01%, precision of 92.02 ± 16.6%, recall of 90.90 ± 14.25%, F1-score of 89.82 ± 14.92%, and Jaccard Index of 84.16 ± 19.91% for the segmentation of PTC in histopathological WSIs. Figure 7 presents the box plots of qualitative evaluation results for the segmentation of PTC. The efficacy and efficiency of the proposed SL-FCN are further evaluated using Fisher’s LSD test (Table 7b). The LSD test results demonstrate that the proposed SL-FCN substantially exceeds the baseline approaches, including U-Net [2] and SegNet [34] in terms of precision, recall, F1-score, and Jaccard index (*p* < 0.001). Furthermore, the qualitative segmentation results of the proposed SL-FCN and the baseline approaches for the segmentation of PTC in Papanicolaou-stained WSIs are presented in Figure 8. A consistency can be seen between the predicted result by the proposed method and the reference standard produced by the expert pathologist in Figure 8.

### 4.6. Ablation Study

In this section, we conduct four experiments to validate the performance of each component of our proposed soft label FCN, including changing the ratio of weight value for different region, changing the soft label regions, utilizing different initialization methods, and utilizing different optimizers with the Kaiming initialization. We conduct the experiments to investigate the soft label regions in our proposed soft label FCN, and analyze the relationships among segmentation performance with our proposed method (see Table 8a). We compare the performance of the proposed soft label FCN with different initialization methods and without initialization (see Table 8b). The quantitative results of the ablation study show that the proposed method without initialization obtains improved performance over the version with Kaiming initialization and Xavier initialization. We compare the performance of the proposed soft label FCN with different ratios of weight which are assigned in different regions (see Table 8c). We also compare the performance of the proposed soft label FCN with Kaiming initialization and different optimizers, including Stochastic Gradient Descent (SGD) with momentum, Adam, Adaptive Gradient, AdaDelta, Nesterov’s Accelerated Gradient (NAG), and RMSprop (see Table 8d). All the experiments are conducted on the DISH dataset 1. The experimental results demonstrate that the proposed method with soft label region $R^{S}$, without initialization, weight values (Ψ=2,Π=1.5,ℵ=1), and SGD with momentum optimizer provides the best performance.

## 5. Discussion and Conclusions

Cancer research has seen constant growth throughout the last few decades. Scientists used several approaches, such as early-stage screening, to detect cancer types before they develop symptoms. Furthermore, they have created novel ways for predicting cancer therapy outcomes early on. However, reliable cancer prediction is one of the most difficult jobs for clinicians. To deal with this challenge, deep learning methods have grown in popularity among medical researchers. The deep learning methods may find and detect patterns as well as accurately determine potential outcomes of a form of cancer. In this study, we develop a SL-FCN method for automated segmentation of HER2 amplification in FISH and DISH images of invasive breast cancer to assist breast cancer target therapy and PTC on Papanicolaou-stained FNA and TP WSIs to help in thyroid cancer diagnosis.

Breast cancer is classified into five subtypes including luminal A, luminal B, HER2-positive luminal B, non-luminal HER2-positive, and triple negative, for treating early breast cancer in the adjuvant setting using levels of ER, PR, Ki67, and HER2 expression [66]. The amplified HER2 gene can be observed in approximately 15–20% of patients with invasive breast cancer as a poor prognostic factor [21,66,67]. HER2 amplification with adverse prognostic effects is not limited to breast and gastric cancer but is also found in a variety of tumor types such as colon cancer, urinary bladder cancer, and biliary cancer [67,68,69,70,71]. Clinical outcomes for HER2–positive breast cancer have dramatically changed with HER2-targeted therapy [21,22]; however, in addition to being expensive, HER2 targeted therapy has some serious side effects associated with its use, such as cardiomyopathy, pulmonary toxicity, and febrile neutropenia [72,73]. Considering these reasons, it is very important to determine the HER2 status for selection of treatment options, and maximizing efficacy while minimizing toxicity and cost is imperative. To date, no biomarkers that predict response to anti-HER2 therapy other than HER2 overexpression itself have been discovered [74]. This requires a reliable method for identifying HER2-positive cases. A key first step in appropriately deciding on the use of HER2-targeted therapy is the accurate determination of HER2 overexpression. IHC detects HER2 protein expression on the cell membrane, and is defined on a scale of 0–3 based on the Hercept Test Score [75]. Scores of 0 and 1+ were considered negative, and a score of 3+ was considered to be positive. An equivocal result, represented by a score of 2+, requires further testing to confirm the presence or absence of HER2 gene amplification, which can be achieved using a second method, most commonly ISH [76]. HER2 ISH was traditionally performed by FISH. DISH provides faster turnaround times and the ability to store slides for long periods without loss of signal [77]. In addition, DISH may also be superior to FISH in assessing heterogeneity, especially when discrete areas of amplification are present within the tumor [78].

The HER2/CEN17 ratio and average HER2 copy number are very important to determine whether the FISH and DISH results are positive or negative. Pathologists rely on their experience to analyze the HER2 gene amplification status of a select region by visual evaluation, which can easily produce bias and inter-observer variability. Therefore, an automated diagnostic method based on AI can potentially overcome the limitations of manual assessment procedure [79,80,81,82]. The development of automated diagnostic tools has been used for segmentation of chromosomes in multicolor FISH images to make pathological examinations more accurate and reliable [30,83,84]. In this study, we developed a soft label FCN technology for analyzing FISH and DISH images. We compared IHC equivocal cases (2+) combined with FISH or DISH testing assessed by visual counting or deep learning methods to confirm HER2 gene status. Using FISH or DISH current standard visual evaluation as a reference, the diagnostic indices for soft label deep learning in (1) FISH dataset with sensitivity 83.52%, specificity 98.65%, and accuracy 93.54%; (2) DISH dataset 1 with sensitivity 91.2%, specificity 86.45%, and accuracy 87.77% and (3) DISH dataset 2 with sensitivity 83.78%, specificity 97.16%, and accuracy 94.64%. Moreover, in statistical analysis, the proposed soft label FCN approach outperforms the baseline approaches by a significant margin (*p* < 0.001). Even for the challenging FISH images with blurry cell borders as shown in Figure 6, the proposed soft label FCN consistently performs well and outperforms benchmark approaches. The approach enables the automated counting of more nuclei with high precision, sensitivity, and accuracy, which is comparable to the usual clinical manual counting method. Adjuvant trastuzumab with chemotherapy is standard treatment for HER2-positive breast cancer, defined as IHC2+ and FISH amplified. Although there is no complete documentation in our experimental data to determine whether FISH-amplified cases are positively associated with treatment outcome, some cases with high HER2 copy number do have a good clinical response that provides oncologists with valuable information on the possibilities of response or not after anti-HER2 target therapy.

PTC is the most common malignant tumor of thyroid cancer. In evaluation for thyroid FNA, pathologists must evaluate all information on glass slides under a light microscope. Digital pathology has emerged as a possible new standard of treatment in recent years, enabling pathology images to be analyzed using computer-based algorithms. However, due to the large size of a typical WSI, pathologists find it difficult to manually detect all of the information in WSI. As a result, artificial intelligence-based automated diagnosis systems are being investigated in order to overcome the limitations of manual and difficult diagnosis procedures. In this study, we developed a soft label FCN technology for analyzing Papanicolaou-stained WSIs for PTC diagnosis. The quantitative evaluation results demonstrate that the proposed method achieves superior performance for the segmentation of PTC on Papanicolaou-stained WSIs than the baseline methods, including Modified FCN, U-Net, and SegNet, with accuracy, precision, and recall of over 90%. Moreover, in statistical analysis based on Fisher’s LSD test, the proposed soft label FCN approach outperforms the baseline approaches, including U-Net and SegNet by a significant margin (*p* < 0.001).

The potential of DL-based soft label approaches in our study have a high degree of accuracy, precision, recall and F1-score. The experimental results on FISH and DISH images of invasive breast cancer for assessment of HER2 amplification and Papanicolaou-stained FNA and TP WSIs for PTC diagnosis demonstrate that the proposed deep learning-based system may not only eliminate misclassification owing to human error, but also decrease the decision-making time, enhancing accuracy and reproducibility while also being more objective, precise, and unbiased than current standard visual interpretation results. People will have more confidence in AI algorithms after they are validated using multi-center data and have increased interpretability. The collaboration between pathologists and AI will promote tumor diagnosis and precision treatment. For live demonstration, an online web-based system of the proposed method has been created. The link of the live demonstration is available in the Supplementary Materials.

## Figures and Tables

**Table 1:** Detailed information of experimental datasets.

Dataset	Overall Magnification	Size (Pixels)	Slides	
DISH breast dataset1	1200×	1600 × 1200	Total	210
			Training	148 (70%)
			Testing	62 (30%)
DISH breast dataset2	600×	1360 × 1024	Total	60
			Training	42 (70%)
			Testing	18 (30%)
FISH breast dataset	600×	1360 × 1024	Total	200
			Training	134 (67%)
			Testing	66 (33%)
FNA and TP thyroid dataset	200×	77,338 × 37,285 (WSI)	Total	131
			Training	28 (21%)
			Testing	103 (79%)

**Table 2:** The structure of the proposed soft label FCN.

Layer	Features (Train)	Features (Inference)	Kernel Size	Stride
Input	512×512×3	512×512×3	-	-
$Conv1_{1}+relu1_{1}$	512×512×3	710×710×64	3×3	1
$Conv1_{2}+relu1_{2}$	710×710×64	710×710×64	3×3	1
Pool1	710×710×64	355×355×64	2×2	2
$Conv2_{1}+relu2_{1}$	355×355×64	355×355×128	3×3	1
$Conv2_{2}+relu2_{2}$	355×355×128	355×355×128	3×3	1
Pool2	355×355×128	178×178×128	3×3	1
$Conv3_{1}+relu3_{1}$	178×178×128	178×178×256	3×3	1
$Conv3_{2}+relu3_{2}$	178×178×256	178×178×256	3×3	1
$Conv3_{3}+relu3_{3}$	178×178×256	178×178×256	3×3	1
Pool3	178×178×256	89×89×256	2×2	2
$Conv4_{1}+relu4_{1}$	89×89×256	89×89×512	3×3	1
$Conv4_{2}+relu4_{2}$	89×89×512	89×89×512	3×3	1
$Conv4_{3}+relu4_{3}$	89×89×512	89×89×512	3×3	1
Pool4	89×89×512	45×45×512	2×2	2
$Conv5_{1}+relu5_{1}$	45×45×512	45×45×512	3×3	1
$Conv5_{2}+relu5_{2}$	45×45×512	45×45×512	3×3	1
$Conv5_{3}+relu5_{3}$	45×45×512	45×45×512	3×3	1
Pool5	45×45×512	23×23×512	2×2	2
$Conv6_{1}+relu6_{1}+drop6$	23×23×512	17×17×4096	7×7	1
$Conv7_{1}+relu7_{1}+drop7$	17×17×4096	17×17×4096	1×1	1
Conv8	17×17×4096	17×17×N	1×1	1
Deconv8	17×17×N	576×576×N	64×64	32
Cropping	576×576×N	512×512×N	-	-
Soft weight loss	512×512×N	512×512×N	-	-
Output	512×512×N	512×512×N	-	-

*N* represents the number of types to predict; in this study *N* = 3, and there are three types to predict, including the background class, the type of tissues other than the targetting type and the targetting tissue type.

**Table 3:** Quantitative evaluation in segmentation of HER2 amplification in each dataset of invasive breast cancer, including (**a**) DISH breast dataset 1; (**b**) DISH breast dataset 2 and (**c**) FISH dataset.

(**a**) DISH Dataset 1						
**Method**	**Accuracy**	**Precision**	**Recall**	**F1-Score**	**Jaccard Index**	**Rank F1-Score**
Proposed soft label FCN	87.77 ± 14.97%	77.19 ± 23.41%	**91.20 ± 7.72%**	**81.67 ± 17.76%**	**72.40 ± 23.05%**	1
U-Net [2] +InceptionV4 [32]	78.74 ± 9.49%	60.48 ± 15.70%	50.67 ± 20.86%	50.88 ± 12.65%	35.10 ± 11.75%	11
Ensemble of U-Net variants ${}^{\iota }$	80.71 ± 9.33%	66.19 ± 17.36%	52.88 ± 20.33%	64.40 ± 12.98%	38.44 ± 12.32%	5
U-Net [2]	80.37 ± 13.38%	63.48 ± 29.03%	3.76 ± 3.86%	6.76 ± 6.35%	3.68 ± 3.59%	14
SegNet [34]	81.89 ± 9.07%	59.06 ± 25.21%	37.38 ± 20.11%	40.20 ± 18.27%	26.78 ± 14.47%	13
Modified FCN [6,7,8,9,10,11]	**91.26 ± 7.56%**	**83.12 ± 11.32%**	71.60 ± 15.38%	75.79 ± 11.39%	62.40 ± 15.43%	2
FCN [36]	81.92 ± 9.43%	51.47 ± 24.20%	50.30 ± 19.18%	48.75 ± 17.78%	34.08 ± 15.45%	12
YOLOv5 [35]	73.19 ± 7.58%	46.38 ± 19.33%	90.38 ± 7.75%	58.22 ± 16.73%	43.22 ± 16.29%	9
DeepLabv3+ [40] with MobileNet [41]	82.76 ± 5.25%	56.56 ± 17.83%	66.74 ± 10.97%	59.20 ± 11.01%	42.43 ± 10.67%	7
DeepLabv3+ [40] with ResNet [33]	82.45 ± 5.90%	55.77 ± 16.42%	62.48 ± 12.84%	56.45 ± 11.39%	39.66 ± 11.10%	10
DeepLabv3+ [40] with Xception [42]	83.53 ± 5.81%	61.74 ± 17.96%	60.72 ± 11.98%	58.93 ± 10.27%	42.04 ± 10.26%	8
CPN [37]	75.94 ± 7.55%	65.94 ± 11.11%	57.13 ± 17.11%	59.37 ± 12.00%	43.21 ± 12.06%	6
SOLOv2 [38]	84.37 ± 6.34%	76.82 ± 7.32%	70.24 ± 15.33%	72.34 ± 10.01%	57.56 ± 11.79%	3
BCNet [39]	83.71 ± 10.15%	76.21 ± 12.40%	62.34 ± 14.30%	67.44 ± 11.08%	51.91 ± 12.45%	4
(**b**) DISH Dataset 2						
**Method**	**Accuracy**	**Precision**	**Recall**	**F1-Score**	**Jaccard Index**	**Rank F1-Score**
Proposed soft label FCN	**94.64 ± 2.23%**	**86.78 ± 8.16%**	**83.78 ± 6.42%**	**85.14 ± 6.61%**	**74.67 ± 10.05%**	1
U-Net [2] +InceptionV4 [32]	84.92 ± 4.31%	73.5 ± 8.11%	65.5 ± 4.54%	67.33 ± 5.23%	50.97 ± 5.92%	5
Ensemble of U-net variants ${}^{\iota }$	84.81 ± 4.38%	74.38 ± 9.55%	61.27 ± 5.81%	66.88 ± 5.84%	51.69 ± 6.95%	6
U-Net [2]	86.89 ± 4.25%	70.39 ± 10.89%	69.09 ± 7.45%	69.12 ± 6.92%	52.97 ± 7.77%	3
SegNet [34]	86.17 ± 3.92%	65.70 ± 10.84%	79.00 ± 8.45%	70.73 ± 5.67%	54.99 ± 6.59%	2
FCN [36]	83.75 ± 5.89%	72.55 ± 10.05%	45.70 ± 12.25%	54.22 ± 9.77%	37.75 ± 8.71%	14
Modified FCN [6,7,8,9,10,11]	89.04 ± 5.26%	82.12 ± 9.48%	59.41 ± 11.96%	68.29 ± 9.98%	52.68 ± 11.51%	4
YOLOv5 [35]	84.66 ± 3.39%	59.77 ± 9.05%	75.05 ± 8.24%	66.38 ± 8.03%	49.61 ± 8.92%	7
DeepLabv3+ [40] with MobileNet [41]	77.33 ± 8.51%	55.06 ± 9.59%	69.50 ± 16.74%	59.78 ± 10.57%	44.00 ± 12.18%	12
DeepLabv3+ [40] with ResNet [33]	80.88 ± 4.56%	59.00 ± 9.15%	73.27 ± 11.80%	64.16 ± 9.19%	48.55 ± 11.99%	9
DeepLabv3+ [40] with Xception [42]	78.72 ± 5.15%	56.00 ± 9.34%	63.61 ± 14.76%	57.88 ± 7.68%	40.66 ± 7.65%	13
CPN [37]	83.61 ± 5.23%	67.39 ± 8.02%	67.22 ± 13.21%	66.33 ± 10.09%	50.33 ± 10.06%	8
SOLOv2 [38]	84.78 ± 6.47%	79.11 ± 10.24%	52.44 ± 7.21%	62.22 ± 5.35%	45.34 ± 5.45%	11
BCNet [39]	83.72 ± 5.74%	73.61 ± 11.42%	57.06 ± 7.18%	63.50 ± 6.40%	48.50 ± 10.85%	10
(**c**) FISH Dataset						
**Method**	**Accuracy**	**Precision**	**Recall**	**F1-Score**	**Jaccard Index**	**Rank F1-Score**
Proposed soft label FCN	**93.54 ± 5.24%**	**91.75 ± 8.27%**	**83.52 ± 13.15%**	**86.98 ± 9.85%**	**78.22 ± 14.73%**	1
Modified FCN [6,7,8,9,10,11]	93.37 ± 4.46%	91.09 ± 7.87%	82.13 ± 10.99%	86.41 ± 8.38%	76.97 ± 12.50%	2
DeepLabv3+ [40] with MobileNet [41]	85.17 ± 5.18%	75.53 ± 6.14%	64.94 ± 9.99%	69.36 ± 7.27%	53.55 ± 8.08%	8
DeepLabv3+ [40] with ResNet [33]	85.06 ± 5.23%	69.78 ± 7.03%	76.44 ± 9.28%	72.52 ± 6.62%	57.29 ± 7.65%	6
DeepLabv3+ [40] with Xception [42]	76.83 ± 11.67%	66.35 ± 19.82%	45.27 ± 24.82%	47.55 ± 20.44%	33.73 ± 15.58%	10
CPN [37]	77.67 ± 8.38%	57.45 ± 8.46%	76.95 ± 8.03%	65.35 ± 6.72%	48.46 ± 7.37%	9
SOLOv2 [38]	88.11 ± 4.48%	79.55 ± 8.01%	75.86 ± 6.6%	77.38 ± 5.82%	62.94 ± 7.45%	5
BCNet [39]	85.98 ± 5.58%	83.27 ± 8.11%	62.36 ± 12.08%	70.55 ± 9.77%	54.80 ± 10.79%	7
Modified mini-U-Net ${}^{\epsilon }$ [61]				83.89%	73.83%	3
mini-U-Net ${}^{\epsilon }$ [62]				81.92%	68.34%	4

${}^{\iota }$ The ensemble model of (**a**) U-Net with Inception-v4 [32]; (**b**) U-Net with Inception-ResNet-v2 encoder [32] and (**c**) U-Net with ResNet-34 encoder [33]. ${}^{\epsilon }$ The evaluation results are referred from [61] on the FISH dataset containing sixteen FISH images with different sizes.

**Table 4:** Statistical analysis to compare the proposed method with benchmark approaches using the LSD test on DISH dataset 1.

LSD Multiple Comparisons							
**Measurement**	**(I) Method**	**(J) Method**	**Mean** **Difference (I-J)**	**Std. Error**	**Sig.**	**95**% **C.I.**	
						**Lower** **Bound**	**Upper** **Bound**
Accuracy	Proposed method	U-Net [2] +InceptionV4 [32]	*** 9.03	1.59	<0.001	5.90	12.15
		Ensemble of U-net variants ${}^{\iota }$	*** 7.06	1.59	<0.001	3.93	10.18
		U-Net [2]	*** 7.40	1.59	<0.001	4.27	10.52
		SegNet [34]	*** 5.88	1.59	<0.001	2.75	9.00
		FCN [36]	*** 5.85	1.59	<0.001	2.72	8.97
		Modified FCN [6,7,8,9,10,11]	* −3.49	1.59	0.029	−6.61	−0.36
		YOLOv5 [35]	*** 14.58	1.59	<0.001	11.45	17.70
		Deeplabv3+ [40] with MobileNet [41]	** 5.01	1.59	0.002	1.89	8.14
		Deeplabv3+ [40] with ResNet [33]	** 5.32	1.59	0.001	2.20	8.45
		Deeplabv3+ [40] with Xception [42]	** 4.24	1.59	0.008	1.12	7.37
		CPN [37]	*** 11.83	1.59	<0.001	8.71	14.96
		SOLOv2 [38]	* 3.40	1.59	0.033	0.28	6.53
		BCNet [39]	* 4.06	1.59	0.011	0.94	7.19
Precision	Proposed method	U-Net [2] +InceptionV4 [32]	*** 16.71	3.37	<0.001	10.10	23.32
		Ensemble of U-net variants ${}^{\iota }$	** 11.00	3.37	0.001	4.37	17.61
		U-Net [2]	*** 13.71	3.37	<0.001	7.10	20.32
		SegNet [34]	*** 18.13	3.37	<0.001	11.52	24.75
		FCN [36]	*** 22.72	3.37	<0.001	16.11	29.34
		Modified FCN [6,7,8,9,10,11]	−5.94	3.37	0.078	−12.55	0.68
		YOLOv5 [35]	*** 30.81	3.37	<0.001	24.19	37.42
		Deeplabv3+ [40] with MobileNet [41]	*** 20.63	3.37	<0.001	14.02	27.24
		Deeplabv3+ [40] with ResNet [33]	*** 24.41	3.37	<0.001	14.81	28.03
		Deeplabv3+ [40] with Xception [42]	*** 15.45	3.37	<0.001	8.84	22.07
		CPN [37]	*** 11.26	3.37	0.001	4.64	17.87
		SOLOv2 [38]	0.37	3.37	0.912	−6.24	6.98
		BCNet [39]	0.98	3.37	0.770	−5.63	7.59
Recall	Proposed method	U-Net [2] +InceptionV4 [32]	*** 40.52	2.70	<0.001	35.23	45.81
		Ensemble of U-net variants ${}^{\iota }$	*** 38.31	2.70	<0.001	33.02	43.60
		U-Net [2]	*** 87.44	2.70	<0.001	82.14	92.73
		SegNet [34]	*** 53.81	2.70	<0.001	48.52	59.10
		FCN [36]	*** 40.89	2.70	<0.001	35.60	46.18
		Modified FCN [6,7,8,9,10,11]	*** 19.59	2.70	<0.001	14.30	24.88
		YOLOv5 [35]	0.81	2.70	0.764	-4.48	6.10
		Deeplabv3+ [40] with MobileNet [41]	*** 24.46	2.70	<0.001	19.15	29.75
		Deeplabv3+ [40] with ResNet [33]	*** 28.71	2.70	<0.001	23.42	34.00
		Deeplabv3+ [40] with Xception [42]	*** 30.47	2.70	<0.001	25.18	35.76
		CPN [37]	*** 34.07	2.70	<0.001	28.78	39.36
		SOLOv2 [38]	*** 20.96	2.70	<0.001	15.66	26.25
		BCNet [39]	*** 28.86	2.70	<0.001	23.57	34.15
F1-score	Proposed method	U-Net [2] +InceptionV4 [32]	*** 30.79	2.38	<0.001	26.11	35.47
		Ensemble of U-net variants ${}^{\iota }$	*** 27.27	2.38	<0.001	22.59	31.95
		U-Net [2]	*** 74.91	2.38	<0.001	70.23	79.59
		SegNet [34]	*** 41.47	2.38	<0.001	36.79	46.15
		FCN [36]	*** 32.92	2.38	<0.001	28.24	37.60
		Modified FCN [6,7,8,9,10,11]	* 5.88	2.38	0.014	1.20	10.57
		YOLOv5 [35]	*** 23.45	2.38	<0.001	18.77	28.13
		Deeplabv3+ [40] with MobileNet [41]	*** 22.47	2.38	<0.001	17.78	27.15
		Deeplabv3+ [40] with ResNet [33]	*** 25.22	2.38	<0.001	20.54	29.90
		Deeplabv3+ [40] with Xception [42]	*** 22.74	2.38	<0.001	18.06	27.42
		CPN [37]	*** 22.30	2.38	<0.001	17.62	26.98
		SOLOv2 [38]	*** 9.34	2.38	<0.001	4.66	14.02
		BCNet [39]	*** 14.24	2.38	<0.001	9.56	18.92
Jaccard Index	Proposed method	U-Net [2] +InceptionV4 [32]	*** 37.30	2.44	<0.001	32.51	42.08
		Ensemble of U-net variants ${}^{\iota }$	*** 33.96	2.44	<0.001	29.18	38.74
		U-Net [2]	*** 68.71	2.44	<0.001	63.93	73.50
		SegNet [34]	*** 45.62	2.44	<0.001	40.84	50.40
		FCN [36]	*** 38.32	2.44	<0.001	33.54	43.10
		Modified FCN [6,7,8,9,10,11]	*** 10.00	2.44	<0.001	5.22	14.78
		YOLOv5 [35]	*** 29.17	2.44	<0.001	24.39	33.96
		Deeplabv3+ [40] with MobileNet [41]	*** 29.96	2.44	<0.001	25.18	34.75
		Deeplabv3+ [40] with ResNet [33]	*** 32.74	2.44	<0.001	27.96	37.52
		Deeplabv3+ [40] with Xception [42]	*** 30.35	2.44	<0.001	25.57	35.13
		CPN [37]	*** 29.19	2.44	<0.001	24.41	33.97
		SOLOv2 [38]	*** 14.84	2.44	<0.001	10.06	19.62
		BCNet [39]	*** 20.49	2.44	<0.001	15.70	25.27

The mean difference is significant at the level of * 0.05, ** 0.01, and *** 0.001. ${}^{\iota }$ The ensemble model of (a) U-Net with Inception-v4 [32]; (b) U-Net with Inception-ResNet-v2 encoder [32]; and (c) U-Net with ResNet-34 encoder [33].

**Table 5:** Statistical analysis to compare the proposed method with benchmark approaches using the LSD test on DISH dataset 2.

LSD Multiple Comparisons							
**Measurement**	**(I) Method**	**(J) Method**	**Mean Difference (I-J)**	**Std. Error**	**Sig.**	**95% C.I.**	
						**Lower Bound**	**Upper Bound**
Accuracy	Proposed method	U-Net [2] +InceptionV4 [32]	*** 9.72	1.72	<0.001	6.33	13.10
		Ensemble of U-net variants ${}^{\iota }$	*** 9.82	1.72	<0.001	6.43	13.21
		U-Net [2]	*** 7.75	1.72	<0.001	4.36	11.13
		SegNet [34]	*** 8.47	1.72	<0.001	5.29	11.64
		FCN [36]	*** 10.89	1.72	<0.001	7.50	14.27
		Modified FCN [6,7,8,9,10,11]	** 5.59	1.72	0.001	2.21	8.98
		YOLOv5 [35]	*** 9.97	1.72	<0.001	6.59	13.36
		Deeplabv3+ [40] with MobileNet [41]	*** 17.31	1.72	<0.001	14.92	20.69
		Deeplabv3+ [40] with ResNet [33]	*** 13.75	1.72	<0.001	10.36	17.14
		Deeplabv3+ [40] with Xception [42]	*** 15.92	1.72	<0.001	12.53	19.30
		CPN [37]	*** 11.03	1.72	<0.001	7.64	14.41
		SOLOv2 [38]	*** 9.86	1.72	<0.001	6.48	13.25
		BCNet [39]	*** 10.92	1.72	<0.001	7.53	14.30
Precision	Proposed method	U-Net [2] +InceptionV4 [32]	*** 13.28	3.21	<0.001	6.96	19.60
		Ensemble of U-net variants ${}^{\iota }$	*** 12.39	3.21	<0.001	6.07	18.71
		U-Net [2]	*** 16.38	3.21	<0.001	10.06	22.70
		SegNet [34]	*** 21.07	3.21	<0.001	14.76	27.39
		FCN [36]	*** 14.22	3.21	<0.001	7.91	20.54
		Modified FCN [6,7,8,9,10,11]	4.66	3.21	0.148	−1.66	10.97
		YOLOv5 [35]	*** 27.00	3.21	<0.001	20.68	33.32
		Deeplabv3+ [40] with MobileNet [41]	*** 31.72	3.21	<0.001	25.41	38.04
		Deeplabv3+ [40] with ResNet [33]	*** 27.78	3.21	<0.001	21.46	34.10
		Deeplabv3+ [40] with Xception [42]	*** 30.78	3.21	<0.001	24.46	37.10
		CPN [37]	*** 19.39	3.21	<0.001	13.07	25.71
		SOLOv2 [38]	* 7.67	3.21	0.018	1.35	13.98
		BCNet [39]	*** 13.17	3.21	<0.001	6.85	19.48
Recall	Proposed method	U-Net [2] +InceptionV4 [32]	*** 21.28	3.45	<0.001	14.48	28.07
		Ensemble of U-net variants ${}^{\iota }$	*** 22.50	3.45	<0.001	15.71	29.30
		U-Net [2]	*** 14.69	3.45	<0.001	7.89	21.48
		SegNet [34]	4.78	3.45	0.167	−2.02	11.57
		FCN [36]	*** 38.07	3.45	<0.001	31.28	44.87
		Modified FCN [6,7,8,9,10,11]	*** 24.36	3.45	<0.001	17.57	31.16
		YOLOv5 [35]	* 8.72	3.45	0.012	1.93	15.52
		Deeplabv3+ [40] with MobileNet [41]	*** 14.28	3.45	<0.001	7.48	21.08
		Deeplabv3+ [40] with ResNet [33]	** 10.50	3.45	0.003	3.71	17.30
		Deeplabv3+ [40] with Xception [42]	*** 20.17	3.45	<0.001	13.37	26.97
		CPN [37]	*** 16.56	3.45	<0.001	9.76	23.35
		SOLOv2 [38]	*** 31.34	3.45	<0.001	24.54	38.13
		BCNet [39]	*** 26.72	3.45	<0.001	19.93	33.52
F1-score	Proposed method	U-Net [2] +InceptionV4 [32]	*** 17.81	2.63	<0.001	12.63	22.99
		Ensemble of U-net variants ${}^{\iota }$	*** 18.25	2.63	<0.001	13.07	23.44
		U-Net [2]	*** 16.01	2.63	<0.001	10.83	21.20
		SegNet [34]	*** 14.40	2.63	<0.001	9.22	19.59
		FCN [36]	*** 30.92	2.63	<.001	25.73	36.10
		Modified FCN [6,7,8,9,10,11]	*** 16.84	2.63	<0.001	11.66	22.03
		YOLOv5 [35]	*** 18.75	2.63	<0.001	13.57	23.94
		Deeplabv3+ [40] with MobileNet [41]	*** 25.37	2.63	<0.001	20.18	30.55
		Deeplabv3+ [40] with ResNet [33]	*** 20.98	2.63	<0.001	15.79	26.16
		Deeplabv3+ [40] with Xception [42]	*** 27.25	2.63	<0.001	22.07	32.44
		CPN [37]	*** 18.81	2.63	<0.001	13.63	23.99
		SOLOv2 [38]	*** 18.81	2.63	<0.001	17.74	28.10
		BCNet [39]	*** 24.64	2.63	<0.001	16.46	26.83
Jaccard Index	Proposed method	U-Net [2] +InceptionV4 [32]	*** 23.70	3.06	<0.001	17.68	29.72
		Ensemble of U-net variants ${}^{\iota }$	*** 22.98	3.06	<0.001	19.96	29.00
		U-Net [2]	*** 21.70	3.06	<0.001	15.68	27.72
		SegNet [34]	*** 19.68	3.06	<0.001	13.66	25.69
		FCN [36]	*** 36.92	3.06	<0.001	30.90	42.94
		Modified FCN [6,7,8,9,10,11]	*** 21.99	3.06	<0.001	15.97	28.01
		YOLOv5 [35]	*** 25.06	3.06	<0.001	19.04	31.08
		Deeplabv3+ [40] with MobileNet [41]	*** 30.67	3.06	<0.001	24.65	36.69
		Deeplabv3+ [40] with ResNet [33]	*** 26.12	3.06	<0.001	20.10	32.14
		Deeplabv3+ [40] with Xception [42]	*** 34.01	3.06	<0.001	27.99	40.03
		CPN [37]	*** 24.35	3.06	<0.001	18.33	30.36
		SOLOv2 [38]	*** 29.33	3.06	<0.001	23.36	35.36
		BCNet [39]	*** 26.17	3.06	<0.001	20.15	32.19

The mean difference is significant at the level of * 0.05, ** 0.01, and *** 0.001. ${}^{\iota }$ The ensemble model of (a) U-Net with Inception-v4 [32]; (b) U-Net with Inception-ResNet-v2 encoder [32]; and (c) U-Net with ResNet-34 encoder [33].

**Table 6:** Statistical analysis to compare the proposed method with benchmark approaches using the LSD test on the FISH dataset.

LSD Multiple Comparisons							
**Measurement**	**(I) Method**	**(J) Method**	**Mean Difference (I-J)**	**Std. Error**	**Sig.**	**95% C.I.**	
						**Lower Bound**	**Upper Bound**
Accuracy	Proposed method	Modified FCN [6,7,8,9,10,11]	0.16	1.17	0.888	−2.13	2.46
		Deeplabv3+ [40] with MobileNet [41]	*** 8.38	1.17	<0.001	6.08	10.67
		Deeplabv3+ [40] with ResNet [33]	*** 8.48	1.17	<0.001	6.19	10.77
		Deeplabv3+ [40] with Xception [42]	*** 16.71	1.17	<0.001	14.42	19.00
		CPN [37]	*** 15.88	1.17	<0.001	13.58	18.17
		SOLOv2 [38]	*** 5.44	1.17	<0.001	3.14	7.73
		BCNet [39]	*** 7.56	1.17	<0.001	5.27	9.85
Precision	Proposed method	Modified FCN [6,7,8,9,10,11]	−0.15	1.76	0.932	−3.60	3.30
		Deeplabv3+ [40] with MobileNet [41]	*** 16.22	1.76	<0.001	12.77	19.68
		Deeplabv3+ [40] with ResNet [33]	*** 21.97	1.76	<0.001	18.51	25.42
		Deeplabv3+ [40] with Xception [42]	*** 25.41	1.76	<0.001	21.95	28.86
		CPN [37]	*** 34.21	1.76	<0.001	30.75	37.66
		SOLOv2 [38]	*** 12.21	1.76	<0.001	8.75	15.66
		BCNet [39]	*** 8.48	1.76	<0.001	5.03	11.93
Recall	Proposed method	Modified FCN [6,7,8,9,10,11]	1.39	2.26	0.538	−3.05	5.83
		Deeplabv3+ [40] with MobileNet [41]	*** 18.59	2.26	<0.001	14.14	23.03
		Deeplabv3+ [40] with ResNet [33]	** 7.09	2.26	0.002	2.64	11.53
		Deeplabv3+ [40] with Xception [42]	*** 38.25	2.26	<0.001	33.81	42.69
		CPN [37]	** 6.57	2.26	0.004	2.13	11.01
		SOLOv2 [38]	** 7.66	2.26	0.002	3.22	12.10
		BCNet [39]	*** 21.16	2.26	<0.001	16.72	25.60
F1-score	Proposed method	Modified FCN [6,7,8,9,10,11]	0.57	1.80	0.752	−2.97	4.11
		Deeplabv3+ [40] with MobileNet [41]	*** 17.61	1.80	<0.001	14.08	21.15
		Deeplabv3+ [40] with ResNet [33]	*** 14.46	1.80	<0.001	10.92	18.00
		Deeplabv3+ [40] with Xception [42]	*** 39.43	1.80	<0.001	35.89	42.97
		CPN [37]	*** 21.63	1.80	<0.001	18.09	25.17
		SOLOv2 [38]	*** 9.60	1.80	<0.001	6.06	13.17
		BCNet [39]	*** 16.43	1.80	<0.001	12.89	19.97
Jaccard Index	Proposed method	Modified FCN [6,7,8,9,10,11]	1.25	1.91	0.515	−2.51	5.00
		Deeplabv3+ [40] with MobileNet [41]	*** 24.67	1.91	<0.001	20.91	28.43
		Deeplabv3+ [40] with ResNet [33]	*** 20.93	1.91	<0.001	17.17	24.69
		Deeplabv3+ [40] with Xception [42]	*** 44.49	1.91	<0.001	40.73	48.25
		CPN [37]	*** 29.75	1.91	<0.001	25.99	33.51
		SOLOv2 [38]	*** 15.27	1.91	<0.001	11.52	19.03
		BCNet [39]	*** 23.41	1.91	<0.001	19.65	27.17

The mean difference is significant at the level of ** 0.01, and *** 0.001.

**Table 7:** Quantitative evaluation with statistical analysis in segmentation of thyroid cancer. (**a**) quantitative evaluation; (**b**) statistical analysis: LSD test.

(**a**)							
**Thyroid Dataset**							
**Method**		**Accuracy**	**Precision**	**Recall**	**F1-Score**	**Jaccard Index**	**Rank F1-Score**
Proposed soft label FCN	ALL	99.99 ± 0.01%	92.02 ± 16.60%	90.90 ± 14.25%	89.82 ± 14.92%	84.16 ± 19.91%	1
	TP	100%	99.86 ± 0.35%	98.35 ± 3.91%	99.06 ± 2.05%	98.22 ± 3.87%	
	FNA	99.99 ± 0.01%	91.36 ± 17.13%	80.28 ± 16.63%	89.04 ± 15.28%	82.98 ± 20.27%	
Modified FCN ${}^{\nu }$ [6,7,8,9,10,11]	ALL	99.99 ± 0.01%	85.91 ± 21.93%	94.39 ± 11.7%	87.6 ± 18.05%	81.6 ± 23.21%	2
	TP	100%	97.03 ± 5.42%	97.85 ± 3.49%	97.41 ± 4.25%	95.12 ± 7.62%	
	FNA	99.99 ± 0.01%	84.97 ± 22.54%	94.10 ± 12.14%	86.78 ± 18.53%	80.45 ± 23.73%	
SegNet ${}^{\nu }$	ALL	92.37 ± 5.99%	81.38 ± 19.11%	55.82 ± 23.45%	61.82 ± 20.79%	47.68 ± 20.04%	4
	TP	97.40 ± 1.59%	97.84 ± 4.6%	56 ± 26.08%	66.95 ± 27.73%	54.86 ± 25.28%	
	FNA	91.95 ± 6.04%	80 ± 19.23%	55.81 ± 23.37%	61.39 ± 20.23%	47.08 ± 19.58%	
U-Net ${}^{\nu }$	ALL	92.14 ± 5.91%	74.03 ± 20.99%	61.03 ± 21.17%	63.68 ± 18.34%	49.21 ± 18.92%	3
	TP	97.42 ± 1.77%	86.72 ± 10.1%	66.26 ± 19.55%	73.68 ± 15.99%	60.34 ± 18.25%	
	FNA	91.7 ± 5.93%	72.96 ± 21.34%	60.59 ± 21.33%	62.84 ± 18.35%	48.27 ± 18.77%	
(**b**)							
**LSD Multiple Comparisons**							
**Measurement**	**(I) Method**	**(J) Method**	**Mean** **Difference** **(I-J)**	**Std. Error**	**Sig.**	**95**%**C.I.**	
						**Lower Bound**	**Upper Bound**
Accuracy	Proposed method	Modified FCN [6,7,8,9,10,11]	<0.01	0.59	0.990	−1.15	1.15
		SegNet [34]	*** 7.62	0.59	<0.001	6.49	8.77
		U-Net [2]	*** 7.88	0.59	<0.001	6.69	9.00
Precision	Proposed method	Modified FCN [6,7,8,9,10,11]	* 6.12	2.75	0.03	0.70	11.53
		SegNet [34]	*** 10.64	2.75	<0.001	5.23	16.05
		U-Net [2]	*** 17.99	2.75	<0.001	12.58	23.41
Recall	Proposed method	Modified FCN [6,7,8,9,10,11]	−3.49	2.55	0.17	−8.50	1.52
		SegNet [34]	*** 35.08	2.55	<0.001	30.07	40.09
		U-Net [2]	*** 29.88	2.55	<0.001	24.86	34.89
F1-score	Proposed method	Modified FCN [6,7,8,9,10,11]	2.21	2.53	0.38	−2.76	7.19
		SegNet [34]	*** 27.99	2.53	<0.001	23.03	32.97
		U-Net [2]	*** 26.14	2.53	<0.001	21.17	31.11
Jaccard Index	Proposed method	Modified FCN [6,7,8,9,10,11]	2.56	2.87	0.37	−3.08	8.20
		SegNet [34]	*** 36.48	2.87	<0.001	30.84	42.12
		U-Net [2]	*** 34.95	2.87	<0.001	29.31	40.59

The mean difference is significant at the level of * 0.05, and *** 0.001. ${}^{\nu }$ The evaluation results are referred from [6] on the thyroid dataset.

**Table 8:** Quantitative results for the ablation study.

(**a**) Quantitative results when changing the soft label regions.					
**Proposed Method**	**Accuracy**	**Precision**	**Recall**	**F1-Score**	**Jaccard Index**
with $R^{S}$ (ϕ = 0.01, υ = 2, τ = 6)	**87.77 ± 14.97%**	77.19 ± 23.41%	**91.20 ± 7.72%**	**81.67 ± 17.76%**	**72.40 ± 23.05%**
with $\frac{1}{2}$$R^{S}$ (ϕ = 0.01, υ = 1, τ = 3)	87.27 ± 13.94%	76.58 ± 22.48%	86.59 ± 10.36%	79.69 ± 17.34%	69.32 ± 21.90%
with $2R^{S}$(ϕ = 0.01, υ = 4, τ = 12)	86.66 ± 10.32%	**79.84 ± 20.09%**	74.80 ± 14.29%	75.62 ± 15.85%	63.17 ± 19.36%
(**b**) Quantitative results when changing the initialization methods.					
**Proposed Method**	**Accuracy**	**Precision**	**Recall**	**F1-Score**	**Jaccard Index**
without initialization	87.77 ± 14.97%	77.19 ± 23.41%	**91.20 ± 7.72%**	**81.67 ± 17.76%**	**72.40 ± 23.05%**
with Kaiming initialization	**89.69 ± 9.93%**	80.37 ± 19.39%	84.08 ± 13.37%	81.16 ± 15.85%	71.02 ± 20.99%
with Xavier initialization	89.36 ± 11.08%	**80.63 ± 20.51%**	84.35 ± 12.70%	81.24 ± 16.64%	71.36 ± 21.59%
(**c**) Quantitative results by modifying the weight parameters of $\omega_{(}m)$: (Ψ,Π,ℵ).					
**Proposed Method**	**Accuracy**	**Precision**	**Recall**	**F1-Score**	**Jaccard Index**
with (Ψ=2, Π=1.5, ℵ=1)	87.77 ± 14.97%	77.19 ± 23.41%	**91.20 ± 7.72%**	**81.67 ± 17.76%**	**72.40 ± 23.05%**
with (Ψ=2, Π=1, ℵ=0.1)	**88.66 ± 10.11%**	**78.83 ± 20.67%**	81.11 ± 14.14%	78.64 ± 16.54%	67.68 ± 21.64%
with (Ψ=4, Π=2, ℵ=1)	87.18 ± 12.82%	78.66 ± 21.13%	84.14 ± 11.71%	79.62 ± 16.19%	68.89 ± 20.90%
(**d**) Quantitative results for the ablation study when using Kaiming initialization and different optimizer.					
**Proposed Method**	**Accuracy**	**Precision**	**Recall**	**F1-Score**	**Jaccard Index**
with SGD with momentum	**89.69 ± 9.93%**	80.36 ± 19.39%	**84.08 ± 13.37%**	**81.16 ± 15.85%**	**71.02 ± 20.99%**
with Adam	63.18 ± 13.30%	34.18 ± 19.90%	21.03 ± 10.18%	22.69 ± 8.59%	13.07 ± 5.68%
with Adaptive Gradient	72.63 ± 11.89%	77.59 ± 23.94%	1.15 ± 1.14%	2.24 ± 2.16%	1.14 ± 1.12%
with AdaDelta	58.87 ± 10.45%	35.39 ± 18.09%	48.87 ± 19.80%	35.02 ± 11.77%	21.88 ± 9.44%
with NAG	87.45 ± 10.54%	79.08 ± 20.31%	79.88 ± 13.46%	77.91 ± 15.81%	66.30 ± 19.70%
with RMSprop	75.05 ± 10.99%	**81.10 ± 13.88%**	14.18 ± 7.15%	23.16 ± 9.14%	13.41 ± 6.22%

## Data Availability

The data that support the findings of this study are available from the corresponding author upon reasonable request.

## Supplementary Materials

An online web-based system of the proposed method has been created for live demonstration. Please see the supplementary video file using this link: https://www.youtube.com/watch?v=eYA_mE6u7EI&ab_channel=ProfChing-WeiWang, accessed on 23 October 2022.
